# Effects of *Salvia mirzayanii* extract administration on hyperglycemia improvement in diabetic rats: The role of GLUT4, PEPCK and G6Pase genes

**DOI:** 10.1016/j.heliyon.2024.e25256

**Published:** 2024-02-01

**Authors:** Rahman Mahdizadehdehosta, Hamid Shahbazmohammadi, Soheila Moein, Neptun Soltani, Kinoosh Malekzadeh, Mahmoodreza Moein

**Affiliations:** a Molecular Medicine Research Center, Hormozgan University of Medical Sciences, Bandar Abbas, Hormozgan, Iran; b Metabolic Diseases Research Center, Research Institute for Prevention of Non-Communicable Diseases, Qazvin University of Medical Sciences, Qazvin, Iran; c Department of Physiology, School of Medicine, Isfahan University of Medical Sciences, Isfahan, Iran; d Department of Medical Genetics, Faculty of Medicine, Hormozgan University of Medical Science, Bandar Abbas, Hormozgan, Iran; e Department of Pharmacognosy, School of Pharmacy, Shiraz University of Medical Sciences, Shiraz, Fars, Iran; f Medicinal Plants Processing Research Center, Shiraz University of Medical Sciences, Shiraz, Fars, Iran

**Keywords:** Diabetes, GLUT4, G6Pase, PEPCK, *Salvia mirzayanii*, Hypoglycemic effect

## Abstract

Diabetes is a dangerous metabolic disorder by increasing incidence in human societies worldwide. Recently, much attention has been focused on the development of hypoglycemic agents, particularly the derivatives of herbal drugs, in the treatment of diabetes. This research aimed to study the anti-diabetic effect of *Salvia mirzayanii* in the diabetic rat models. First, the plant material was extracted from the leaves, and orally administered to the rats. After treating the animals with the aqueous extract of *S. mirzayanii* at a dose of 600 mg/kg, animal body weight for 12 weeks, fasting blood glucose, oral glucose tolerance test (OGTT), and body weight changes were examined. To analyze the anti-diabetic function of *S. mirzayanii*, we measured the expression of **glucose transporter-4** (GLUT4), phosphoenolpyruvate carboxykinase (PEPCK), and glucose 6-phosphatase (G6Pase) genes in healthy and streptozotocin (STZ)-diabetic rats. The expression levels of the genes of interest in muscle and liver tissues were determined using reverse transcription-quantitative polymerase chain reaction (RT-qPCR) and immunohistochemistry (IHC). There were no significant differences in fasting blood glucose and OGTT between normal control (NC) group and the diabetic control (DC) group treated with *S. mirzayanii*. In contrast, there was a significant difference with the untreated DC (*P < 0.05*). The treatment of diabetic rats with *S. mirzayanii* significantly increased the expression of GLUT4 in the muscle and decreased the expression levels of PEPCK and G6Pase in the liver compared to the DC group (*P < 0.05*). These findings clearly show that *S. mirzayanii* can improve hyperglycemia by increasing the GLUT4 expression, and inhibiting the gluconeogenesis pathway in the liver. In general, the obtained results provided a new insight into the efficacy of *S. mirzayanii* aqueous extract as an anti-diabetic herbal medicine.

## 1 Introduction

Diabetes is a chronic health condition by increasing incidence in human societies worldwide. Diabetes mellitus is a metabolic syndrome disorder characterized by the persistent hyperglycemia and hyperlipidemia. The primary causes of morbidity and death are linked to it: neuropathy, nephropathy, retinopathy, cardiovascular, and cerebrovascular problems. The increasing incidence of diabetes in children and young people, along with the associated morbidity, mortality, and growing racial and ethnic disparities, is a major public health challenge [1,2]. Based on research published in the Lancet, new estimates predict that the number of people with diabetes will reach nearly more than 1.3 billion people worldwide by the year 2050, up from about 529 million in 2021 [3]. Patients are usually treated with anti-diabetic agents, such as insulin and metformin. However, some of these drugs have limited effectiveness and various side effects, including drug tolerance, headache, megaloblastic anemia, dermatitis, acidosis, etc. Therefore, searching for safer and more effective hypoglycemic pharmaceuticals is of utmost interest [[4], [5], [6], [7]].

Medicinal plants have emerged as effective sources of anti-diabetic medicines in recent years, serving as complimentary or alternative options to conventional drugs. Herbal medicines with anti-diabetic potential acting via their insulin-mimetic properties could inhibit intestinal absorption of glucose or insulin-dependent metabolic processes [[8], [9], [10]]. Natural compounds, such as alkaloids, peptidoglycans, terpenoids, amino acids, and inorganic ions have hypoglycemic effects. The hypoglycemic activity of many medicinal plants has been evaluated and confirmed in different animal models [11,12]. For example, *Salvia* is the largest genera of the Lamiaceae family, containing over 900 species worldwide that demonstrate different therapeutic activities to treat various diseases. The members of genus *Salvia* have important biological and pharmacological properties, e.g., astringent, antiseptic, spasmolytic, and anti-inflammatory effects [[13], [14], [15], [16], [17], [18]]. Various species of *Salvia* were used in conventional medicine to treat diabetes [16,18]. In folk Iranian medicine, *Salvia mirzayanii* (locally called Bitter Moor) is a perennial plant that grows in southern and central parts of Iran. The extract of *S. mirzayanii* leaves was suggested for diabetes, and its antioxidant activity shows this effect [[17], [18], [19]]. The hypoglycemic activity of *S. mirzayanii* in diabetic patients was reported by Moein et al. [19]. Javid et al. indicated the significant inhibitory effect of *S. mirzayanii* extract on α-amylase enzyme as a diabetes biomarker [20]. Given the widespread usage of S. mirzayanii in traditional medicine to treat diabetes, and the limited number of studies on its anti-diabetic effects, it is crucial to do more research using molecular function analysis. This study aimed to study the effects of the aqueous extract of *S. mirzayanii* leaves on the expression level of glucose transporter-4 (GLUT4), phosphoenolpyruvate carboxykinase (PEPCK), and glucose 6-phosphatase (G6Pase) genes in streptozotocin (STZ)-induced diabetes in rat models. The choice of solvent for the extraction of medicinal plants depends on the type of plant, the part of plant to be extracted, and the nature of bioactive compounds. As we know, water is the most polar solvent, and is used to extract a wide range of polar compounds [21]. Hence, various factors, such as solubility of compounds of *S. mirzayanii* extract, safety of administration to animals as well as cost were considered when choosing water as the extraction solvent. Thus, we aimed to study the molecular action of *S. mirzayanii* relating to diabetes mellitus for further research and drug development.

## 2 Materials and methods

### 2.1 Chemicals and kits

DEPC water and agarose gel was purchased from Cinna Gen Company (Tehran, Iran). RNA extraction kit and reverse transcription-quantitative polymerase chain reaction (RT-qPCR) kits were procured from QIAGEN Company (Germany). Primers were synthesized by Bioneer Company (Daejon, Korea). All other chemicals were prepared from Merck (Germany), and Sigma (St Louis, MO, USA).

### 2.2 Preparation of *S. mirzayanii* aqueous extract

*S. mirzayanii* grows widely in Bandar-Abbas province (Iran). The voucher number assigned to S. mirzayanii is MPRCM-94-84, which has been deposited with the Medicinal Plants Processing Research Center at the School of Pharmacy, Shiraz University of Medical Sciences. To prepare the aqueous extract of *S. mirzayanii*, 100 g of dried leaves obtained from 130 g of the raw plant was initially boiled in 1 L of distilled water for 10 min and then maintained at 70 °C at Ben Marie (Memmert GmbH, Germany) for 24 h. The aqueous extract was filtered with Whattman filter paper No 1, and dried at 50 °C. The extracted substance (20 g per 100 g of crushed plant material) was combined and thinned with normal saline to reach a targeted concentration (600 mg/mL) before being filtered. The extract was kept at a temperature of 4 °C until it was utilized for studies [22,23].

### 2.3 Animals

Male Wistar rats at ages 6–9 weeks (200–250 g), and their foods were purchased from the Pasteur Institute of Iran, Karaj, Iran. They were housed in an air-controlled room at 12 h: 12 h light-dark cycle supplied with **a pellet diet and filtered tap water.** The ingredients of pellet diet include 23.0 % crude protein, 10.0 % moisture, 0.5 % sodium chloride, 1.0 % calcium, 0.65 % phosphorus, 0.25 % tryptophan, 0.33 % methionine, 1.15 % lysine, 0.7 % threonine, 4.0 % crude fat and 4.0 % crude fiber. **Prior to the commencement of the experiment, a one-week period was** granted **for them to acclimate to the laboratory environment.** The rats were divided based on their body weight into three groups (n = 5) as follows: 1- Normal control (NC) received a normal diet. 2- Diabetic control (DC) received 1 mL of normal saline. 3- Diabetic rats were treated orally with an aqueous extract of *S. mirzayanii* (600 mg/kg) for 12 weeks [19]. The animals were fasted overnight (for about 12 h) before the induction of anesthesia [24]. This study was confirmed by the ethics committee of Qazvin University of Medical Sciences (Ethical code# IR.QUMS.AEC.1402.006).

### 2.4 Induction of diabetes

Before the administration of STZ, animals were fasted but had free access to water. The body weight of rats was taken, and the average body weight in NC, DC, and treatment groups were 240, 230, and 239 g, respectively, Diabetes was induced by a single intraperitoneal injection of STZ solution (50 mg/kg body weight) [16,25]. Control animals received an injection with normal saline. After 48 h of the induction of diabetes, fasting blood sugar (FBS) levels were measured with a **glucometer** (Easy Gluco, Infopia Co., Ltd., South Korea). Rats with FBS levels above 220 mg/dl were considered diabetic. Diabetic rats exhibited symptoms of frequent urination and polydipsia.

### 2.5 Evaluation of FBS, oral glucose tolerance test (OGTT) and animal weight

All animal groups were fasted overnight before blood collection which had free access to water. FBS values were measured **using** Easy Gluco during the study and the day before diabetes induction. OGTT, in which the rats are challenged with a glucose bolus, was performed across a 2-h time course based on the standard protocol [25]. Prior to the test, the rats were fasted for 16 h, and blood glucose levels were monitored from the tail, and blood glucose levels were monitored from the tail tip using Easy Gluco. The rats then received 2.5 g/kg body weight of a 100 mg/mL glucose solution in sterile water delivered by oral gavage. At 10, 20, 30, 60, 90, and 120 min after administering glucose, blood was collected from the tail to measure the glucose concentration using Easy Gluco. The change in body weight of animals was regularly recorded during the experiment period.

### 2.6 Preparation of tissue samples

All rats were anesthetized using a mixture of ketamine (50.0 mg/kg) and xylazine (5.0 mg/kg). Following complete anesthesia, the animals were subjected to a wide laparotomy under aseptic conditions. In brief, the rats were placed resting on the surgery table, and then the abdominal skin was impregnated with a betadine solution of 10 %. The abdominal hairs were entirely severed with a straight razor, rendering the skin prepared for surgery. A midline abdominal incision was performed with a knife, and sections of the hepatic tissue were excised. To isolate the gastrocnemius muscle, an incision was made at the distal end of thigh, and the beginning of the leg, and the muscle was removed. Afterwards, the prepared samples were washed with cold normal saline 0.9 % and placed at −80 °C in an RNA buffer solution for subsequent experiments. Finally, to ensure the death of animals, they were beheaded with a guillotine under complete anesthesia [24].

### 2.7 Expression assessment of GLUT4, PEPCK and G6Pase

To study the effects of *S. mirzayanii* extract, mRNA expression measurements of GLUT4, PEPCK, and G6Pase were performed by RT-qPCR. The primers of GLUT4، PEPCK, G6Pase and β-actin were designed using DNASIS MAX 3.0 software (DNASIS version 3.0, Hitachi Software Engineering Co., Ltd., Tokyo, Japan) (Table 1). The β-actin gene was used as an internal standard (housekeeping gene). Each group's frozen liver and muscle samples were thawed and used for RNA extraction. The purity of RNA at 260/280 OD ratio and RNA integrity were evaluated and only samples of high purity (OD 260/280 > 1.9) were subjected to further manipulation. RT-qPCR was performed using the QuantiNova SYBR Green RT-qPCR Kit (QIAGEN, Germany). Results were normalized using the equation 2^−ΔΔCt^.

**Table 1:** Primer sequences and expected ampliocn size of the genes for RT-qPCR analysis.

Gene	(5′→3′) Sequence	Length (mer)	Tm (°C)	GC%	Product Size (Base pair)
GLUT4	Forward: ACAATGTCTTGGCTGTGCTG	20	55.2	50	140
	Reverse: TCCCACATACATAGGCACCA	20	55.4	50	
PEPCK	Forward: CATTACCCAAGAGCAGAGAG	20	51.2	50	151
	Reverse: GAATGGGATGACATACATGG	20	51.4	45	
G6Pase	Forward: TTCCGGTGCTTGAATGTCGT	20	60.0	50	79
	Reverse: GCAAGGTAGATCCGGGACAG	20	58.4	60	
β-actin	Forward: ACGGTCAGGTCATCACTATC	20	50.1	50	228
	Reverse: AGAGGTCTTTACGGATGTCA	20	50.6	45	

GLUT4 = glucose transporter 4; PEPCK = phosphoenolpyruvate carboxy kinase; G6Pase = glucose 6-phosphatase; Tm = Temperature of melting; GC = Guanine cytosine.

### 2.8 Immunohistochemistry (IHC) test

Five-micrometer sections of formalin-fixed paraffin-embedded liver and muscle tissue were cut and prepared for IHC staining. The tissues underwent xylene processing and a graded alcohol series, followed by treatment with 3.0 % H2O2 to remove endogenous peroxidase activity before immunological staining. Subsequently, they were blocked with 5.0 % normal goat serum. Slides were then treated with mouse anti-rat GLUT4 mAb (Cell Signaling Technology, USA), anti-rat G6Pase polyclonal antibody (Biorbyt Ltd, United Kingdom) and mouse anti-rat PEPCK mAb (Santa Cruz Biotechnology, Santa Cruz, CA, USA) at a 10 μg/mL concentration. After overnight incubation, horse anti-mouse IgG biotin secondary antibody (Vector Labs, Burlingame, CA) was added at 1:200 dilutions, followed by streptavidin–HRP (Jackson Labs, West Grove, PA) at a 1:1000 dilution. Slides were developed with AEC reagent (Zymed Laboratories, San Francisco, CA, USA) and counterstained with hematoxylin (Sigma Co, St. Louis, MO). Cells were viewed and photographed under a bright field microscope [25].

### 2.9 Statistical tests

All tests were carried out by GraphPad Prism version 7.00 (GraphPad Software, La Jolla California USA). Data were presented as mean ± standard deviation and statistically analyzed using one-way ANOVA. Differences with *p*-values below 0.05 were considered significant.

## 3 Results

### 3.1 Effect of *S. mirzayanii* on FBS, OGTT and body weight

**Fig. 1:**
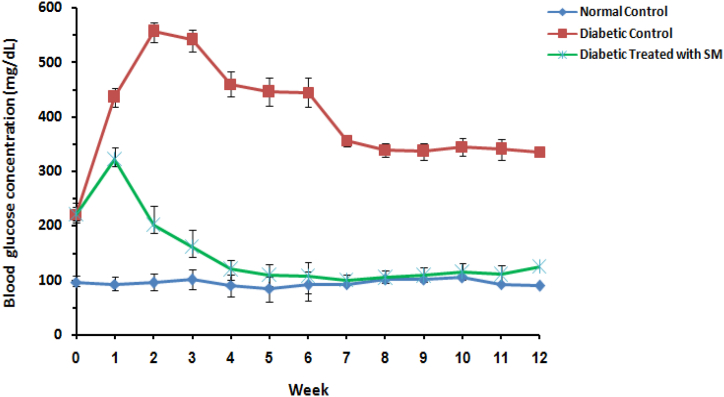
Variation of blood glucose concentration of rats during 12 weeks in normal control (NC), diabetic control (DC) and diabetic group treated with *S. mirzayanii* (SM) aqueous extract.

**Fig. 2:**
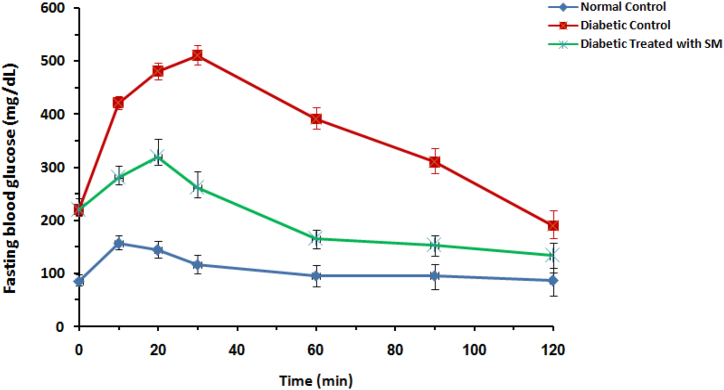
Variation of fasting blood glucose of rats in glucose tolerance test in normal control (NC) group, diabetic control (DC) and diabetic group treated with *S. mirzayanii* (SM) aqueous extract.

**Fig. 3:**
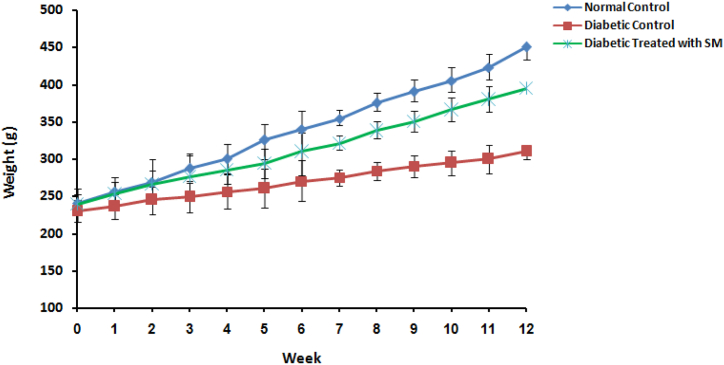
Variation of body weight of rats during 12 weeks in normal control group (NC), diabetic control (DC) and diabetic group treated with *S. mirzayanii* (SM) aqueous extract.

Firstly, to study the effects of *S. mirzayanii* extract in rat models, FBS levels, OGTT and body weight changes were measured. Over a period of 12 weeks, the administration of S. mirzayanii extract prevented the rise in FBS level in the diabetic group that received treatment, as compared to the DC group (Fig. 1). OGTT results in the diabetic group treated with *S. mirzayanii* indicated a significant reduction in glucose levels compared to that of DC group (Fig. 2). The results of body weight measurements in rats showed smaller changes in the diabetic group treated with *S. mirzayanii* than in the DC group (Fig. 3).

### 3.2 Molecular function analysis of *S. mirzayanii*

To study the molecular functions of *S. mirzayanii* extract, mRNA from liver and muscle tissues was subjected to RT-qPCR analysis. GLUT4, PEPCK, and G6Pase gene expression levels were evaluated in NC, DC, and diabetic rats treated with *S. mirzayanii* aqueous extract.

### 3.3 Effect of *S. mirzayanii* on expression of GLUT4

**Fig. 4:**
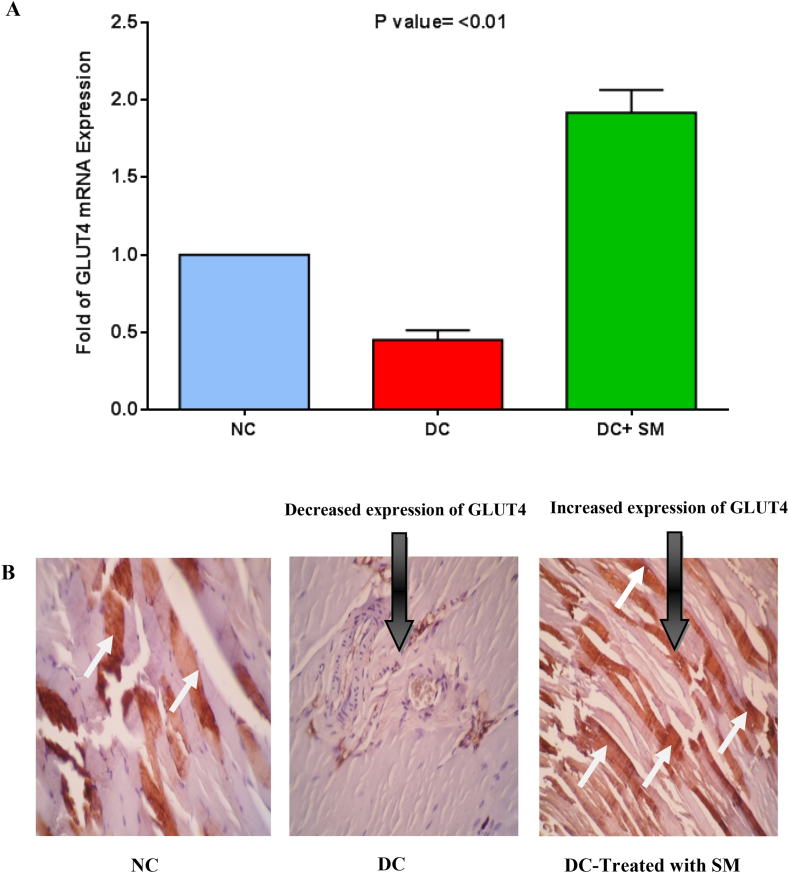
Analysis of GLUT4 expression in the muscle tissue in normal control (NC), diabetic control (DC) and diabetic control treated with *S. mirzayanii* (DC-Treated with SM). (A) Analysis of mRNA expression using RT-qPCR. Data were normalized with that of β-actin and then calculated as relative to the NC group. Each value represents the mean ± SEM (n = 10 per group) in two independent experiments. *P* < 0.01: Significant difference vs. control group. (B) Protein expression level analysis by IHC technique in NC, DC and DC-Treated with SM. The small white arrows indicate the expression of GLUT4 in the muscle tissue.

The mRNA levels of GLUT4 in the muscle tissue of all groups were analyzed. The expression level of GLUT4 mRNA in the NC group was considered a reference to calculate the other groups. Treatment of diabetic rats with *S. mirzayanii* aqueous extract elevated GLUT4 mRNA expression when compared with DC group (Fig. 4A). RT-qPCR results showed that *S. mirzayanii* administration in diabetic rats induces a significant increase (*P* < 0.01) in the GLUT4 expression (Please see more details in Table 1 of Supplementary file). Immunohistochemical (IHC) labeling was performed to examine the expression level of the GLUT4 protein in muscle tissue. The findings are shown in Fig. 4B. The results clearly demonstrated that the diabetic rats treated with S. mirzayanii exhibited an increase in GLUT4 protein levels in their muscles. However, this effect was not seen in the DC group.

### 3.4 Effect of *S. mirzayanii* on expression of PEPCK and G6Pase

**Fig. 5:**
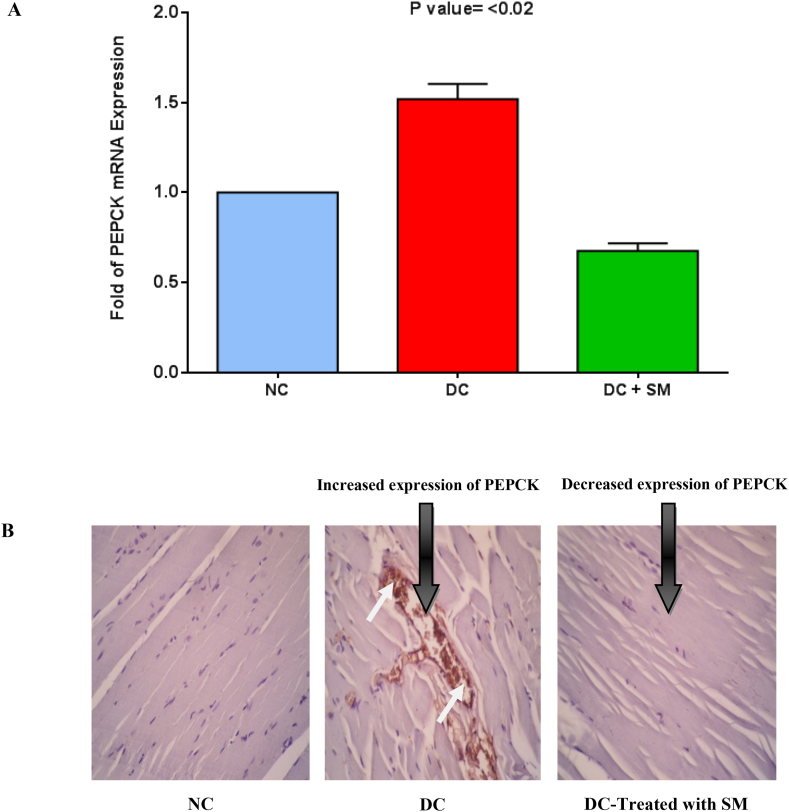
Analysis of PEPCK expression in the liver tissue in normal control (NC), diabetic control (DC) and diabetic control treated with *S. mirzayanii* ((DC-Treated with SM). (A) Analysis of mRNA expression using RT-qPCR. Data were normalized with that of β-actin and then calculated as relative to the NC group. Each value represents the mean ± SEM (n = 10 per group) in two independent experiments. *P* < 0.02: Significant difference vs. control group. (B) Protein expression level analysis by IHC technique in NC, DC and DC-Treated with SM. The small white arrows indicate the expression of PEPCK in the liver tissue.

**Fig. 6:**
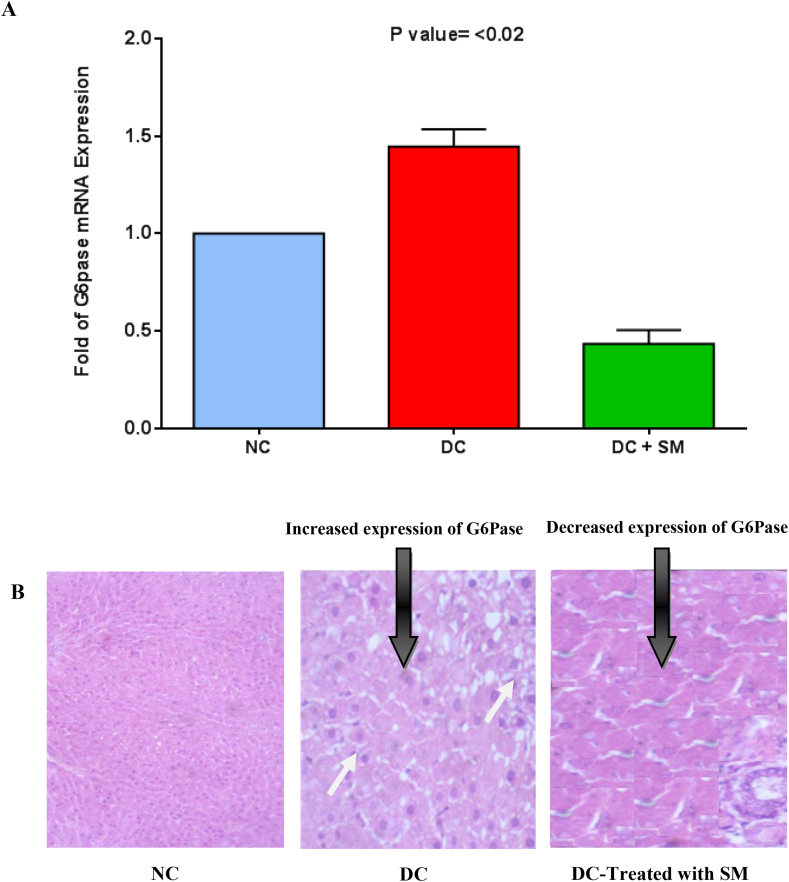
Analysis of G6Pase expression in the liver tissue in normal control (NC), diabetic control (DC) and diabetic control treated with *S. mirzayanii* (SM). (A) Analysis of mRNA expression using RT-qPCR. Data were normalized with that of β-actin and then calculated as relative to the NC group. Each value represents the mean ± SEM (n = 10 per group) in two independent experiments. *P* < 0.02: Significant difference vs. control group. (B) Protein expression level analysis by IHC technique in NC, DC and DC-Treated with SM. The small white arrows indicate the expression of G6Pase in the liver tissue.

The expression of PEPCK and G6Pase genes was determined when the diabetic rats were treated with *S. mirzayanii* aqueous extract. After treatment with *S. mirzayanii*, the expressions of PEPCK and G6Pase mRNAs were significantly decreased (*P* < 0.02) compared to NC and DC rats (Fig. 5, Fig. 6A) (Please see more details in Tables 2 and 3 of Supplementary file). Immunohistochemistry (IHC) was conducted to examine the expression levels of PEPCK and G6Pase proteins in the liver. The findings are shown in Fig. 5, Fig. 6B. The findings of our study clearly demonstrated a substantial reduction in the protein levels of PEPCK and G6Pase in the liver of diabetic rats treated with *S. mirzayanii*, as compared to the NC and DC groups.

## 4 Discussion

Diabetes is a persistent metabolic disorder that affects the global population. Acquiring knowledge and being conscious of diabetes, including its risk factors, complications, and treatment, are crucial elements for achieving better control and enhancing the quality of life. For these reasons, there is an increasing interest in hypoglycemic agents derived from natural products, especially plant materials. Therefore, it is essential to find better biologically active ingredients from herbs and natural products to treat diabetes [26,27].

In recent years, numerous plant extracts and formulations were examined to regulate the expression of genes in the metabolic pathways of diabetic animal models. The alteration in the manifestation of associated genes is a crucial element in the development of diabetes. Various species of *Salvia* are applied in traditional medicine to treat diabetes [28,29]. Among the family of *Salvia*, *S. mirzayanii* herb is of particular interest because of its pharmaceutical features. In folk Iranian medicine, *S. mirzayanii* leaves were used for stomach pains [30]. Furthermore, previous studies have reported the hypoglycemic effect and anti-hyperlipidemic potency of *S. mirzayanii* in diabetes in rats, while the action mode of this plant has not been reported yet. In this communication, we evaluated the effect of *S. mirzayanii* extract on GLUT4 as a glucose transporter and two key enzymes of carbohydrate metabolism (PEPCK and G6Pase) at the mRNA and protein levels of diabetic rats using RT-qPCR and IHC techniques.

First, to evaluate the effect of *S. mirzayanii*, FBS levels, glucose tolerance, and body weight changes were studied. During 12 weeks of administration, *S. mirzayanii* extract decreased FBS levels in the diabetic rats. The results of OGTT in the rats treated with *S. mirzayanii* showed a reduction in glucose levels compared to the DC group. Body weight measurements revealed fewer changes in the diabetic group treated with *S. mirzayanii* than in the DC group. As preliminary data, these results mean that *S. mirzayanii* has an anti-diabetic effect. These data agreed with the findings about *S. mirzayanii* [18,19,31]. The hypoglycemic effects for the other species of *salvia* genus like *S. hydrangea* [32], *S. officinalis* [14,16], *S. sahendicahas* [33], *S. miltiorrhiza* [13,34] have also been reported in previous studies.

In order to better analyze the molecular function of *S. mirzayanii*, the effect of this herb on the expression of GLUT4, PEPCK, and G6Pase was studied. GLUT4 is a transmembrane carrier protein that allows glucose to move through cell membranes. It is the main transporter for transferring glucose between muscle and blood and the reabsorption of glucose in the kidney. GLUT4 mRNA and protein expression levels after administrating *S. mirzayanii* aqueous extract increased. This finding means that *S. mirzayanii* increases GLUT4 expression to induce effects like insulin. Then, the expression of PEPCK and G6Pase in the liver was investigated. After treating the diabetic rats with *S. mirzayanii*, mRNAs and proteins expression of PEPCK and G6Pase were significantly decreased. These findings suggest that the anti-hyperglycemic action of *S. mirzayanii* is likely to be associated with the up-regulation of GLUT4 in muscle and down-regulation of PEPCK and G6Pase expressions in the liver as documented by RT-qPCR and IHC studies. The radical scavenging activity and reducing power for *S. mirzayanii* was shown in the literature [35]. *Salvia* spp. The major components are comprised of many biologically active compounds, e.g., polyphenols, flavonoids, terpenoids, and salvianolic acids [36,37]. The reports of other works have showed that the anti-hyperglycemic property of *S. mirzayanii* is in terms of the presence of compounds, such as polyphenols and flavonoids. These compounds have hypoglycemic properties and work by inhibiting the carbohydrate hydrolyzing enzymes, including α-amylase and α-glucosidase [38,39]. One of the important functions of polyphenols is the inhibition of α-amylase and α-glucosidase. **Indeed, a thorough investigation combining bioinformatics and experimental study is necessary to identify the specific bioactive compounds responsible for the hypoglycemic effects of this herbal plant.**

## 5 Conclusion and recommendations

In this work, the aqueous extract of *S. mirzayanii* leaves significantly reduced hyperglycemia by increasing the expression of GLUT4, and decreasing the expression levels of PEPCK and G6Pase. It can be concluded that *S. mirzayanii* extract affects the genes related to glucose metabolism via up-regulation of GLUT4 and down-regulation of PEPCK and G6Pase expressions.

The findings of this research indicate that *S. mirzayanii* can be proposed as a herbal medicine for treating hyperglycemia and diabetes, but a conclusion should be made after further studies. Indeed, additional investigation is required to clarify the precise mechanism of action of *S. mirzayanii* and to identify the primary active ingredients accountable for the noted hypoglycemic effect. This will constitute one of our goals for plant-based drug discovery in diabetes mellitus in the next work. Finally, we suggest that clinical trial tests are performed to evaluate whether such therapy can be administered in the diabetic population.

## Funding statement

This research was funded by the 10.13039/501100011917Hormozgan University of Medical Sciences of Iran and the, 10.13039/501100006396Qazvin University of Medical Sciences of Iran.

## Data availability statement

Data included in article/supplementary material/referenced in article.

## CRediT authorship contribution statement

**Rahman Mahdizadehdehosta:** Investigation, Conceptualization. **Hamid Shahbazmohammadi:** Writing – review & editing, Writing – original draft, Validation, Methodology. **Soheila Moein:** Supervision, Funding acquisition. **Neptun Soltani:** Validation, Resources. **Kinoosh Malekzadeh:** Visualization, Investigation. **Mahmoodreza Moein:** Visualization, Software, Data curation.

## Declaration of competing interest

The authors declare the following financial interests/personal relationships which may be considered as potential competing interests:Hamid Shabazmohammadi reports financial support was provided by 10.13039/501100006396Qazvin University of Medical Sciences. Hamid shahbazmohammadi reports a relationship with Qazvin University of Medical Sciences that includes: employment. Hamid shahbazmohammadi has patent pending to licensee. Rahman Mahdizadehdehosta, Hamid Shahbazmohammadi, Soheila Moein, Neptun Soltani, Kinoosh Malekzadeh, Mahmoodreza Moein If there are other authors, they declare that they have no known competing financial interests or personal relationships that could have appeared to influence the work reported in this paper.
