# Supplementary material for: Effects of Salvia mirzayanii extract administration on hyperglycemia improvement in diabetic rats: The role of GLUT4, PEPCK and G6Pase genes
Source: Heliyon. 2024 Feb 1;10(3):e25256. doi: 10.1016/j.heliyon.2024.e25256 (PMC10850551; doi:10.1016/j.heliyon.2024.e25256)
Supplement: Multimedia component 1 [file mmc1.doc]

**Supporting Information**

**Effects of *Salvia mirzayani***i extract **administration on hyperglycemia improvement in diabetic rats: The role of GLUT4, PEPCK and** **G6Pase** genes

Rahman Mahdizadehdehosta ^a^, Hamid Shahbazmohammadi ^b,*^, Soheila Moein ^a, **^, Neptun Soltani ^c^, Kinoosh Malekzadeh ^d^, Mahmoodreza Moein ^e, f^

*^a^ Molecular Medicine Research Center, Hormozgan University of Medical Sciences, Bandar*

*Abbas, Hormozgan, Iran*

*^b^ Metabolic Diseases* *Research Center, Research Institute for Prevention of Non-Communicable Diseases,* Qazvin University of Medical Sciences**,** Qazvin, Iran

*^c^ Department of Physiology, School of Medicine, Isfahan University of Medical Sciences, Isfahan, Iran*

*^d^ Department of Medical Genetics, Faculty of Medicine, Hormozgan University of Medical Science, Bandar Abbas, Hormozgan, Iran*

*^e^ Department of Pharmacognosy, School of Pharmacy, Shiraz University of Medical Sciences, Shiraz, Fars, Iran*

*^f^ Medicinal Plants Processing Research Center, Shiraz University of Medical Sciences, Shiraz, Fars, Iran*

^*^ Corresponding author.

^* *^ Corresponding author.

*E-mail addresses:* [h.shahbazmohammadi@qums.ac.ir](mailto:h.shahbazmohammadi@qums.ac.ir) (H. Shahbazmohammadi), mpcd@fct.unl.pt (S. Moein)

**Table 1.** RT-qPCR results for expression analysis of GLU-T4.

| 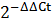 | ΔΔC_T_ | ΔC_T_ | Average C_T_  GLU-T4 β-actin | | Sample | Group |
| --- | --- | --- | --- | --- | --- | --- |
|  |  | 6.65 | 12.52 | 19.17 | 1 | Normal control |
|  |  | 6.23 | 12.99 | 19.22 | 2 |
|  |  | 8.55 | 12.77 | 21.32 | 3 |
|  |  | 6.80 | 14.34 | 21.14 | 4 |
|  |  | 5.98 | 15.47 | 21.45 | 5 |
| **1.0** | **0** | **6.84** | **13.61** | **20.86** |  | Average |
|  |  | 8.18 | 13.78 | 21.95 | 1 | Diabetic Control |
|  |  | 8.26 | 12.88 | 22.08 | 2 |
|  |  | 8.36 | 13.76 | 22.11 | 3 |
|  |  | 8.47 | 13.62 | 21.09 | 4 |
|  |  | 7.36 | 13.76 | 21.18 | 5 |
| **0.42** | **1.28** | **8.12** | **13.56** | **21.68** |  | Average |
|  |  | 5.77 | 13.47 | 19.38 | 1 | Diabetic control treated with SM |
|  |  | 5.92 | 12.91 | 19.45 | 2 |
|  |  | 5.95 | 13.75 | 19.47 | 3 |
|  |  | 6.00 | 13.62 | 19.38 | 4 |
|  |  | 5.98 | 13.81 | 19.48 | 5 |
| **1.90** | **-0.92** | **5.92** | **13.51** | **19.43** |  | Average |

**Table 2.** RT-qPCR results for expression analysis of PEPCK.

| 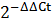 | ΔΔCT | ΔCT | Average C_T_  PEPCK β-actin | | Sample | Group |
| --- | --- | --- | --- | --- | --- | --- |
|  |  | 12.11 | 12.52 | 24.11 | 1 | Normal control |
|  |  | 11.66 | 12.99 | 23.41 | 2 |
|  |  | 11.51 | 12.77 | 23.28 | 3 |
|  |  | 9.74 | 14.34 | 23.08 | 4 |
|  |  | 11.72 | 14.87 | 23.18 | 5 |
| **1.0** | **0** | **9.92** | **13.49** | **23.41** |  | Average |
|  |  | 9.30 | 13.52 | 22.42 | 1 | Diabetic Control |
|  |  | 9.77 | 13.99 | 22.96 | 2 |
|  |  | 9.00 | 13.79 | 22.89 | 3 |
|  |  | 8.11 | 12.34 | 22.87 | 4 |
|  |  | 9.55 | 13.87 | 22.86 | 5 |
| **1.53** | **-0.62** | **9.30** | **13.50** | **22.80** |  | Average |
|  |  | 11.61 | 13.47 | 23.88 | 1 | Diabetic control treated with SM |
|  |  | 11.03 | 13.42 | 24.05 | 2 |
|  |  | 11.32 | 13.75 | 24.16 | 3 |
|  |  | 10.42 | 13.62 | 24.14 | 4 |
|  |  | 11.24 | 13.81 | 24.12 | 5 |
| **0.67** | **0.56** | **10.48** | **13.61** | **24.00** |  | Average |

**Table 3.** RT-qPCR results for expression analysis of G6pase.

| 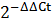 | ΔΔC_T_ | ΔC_T_ | Average C_T_  G6pase β-actin | | Sample | Group |
| --- | --- | --- | --- | --- | --- | --- |
|  |  | 12.11 | 12.52 | 24.63 | 1 | Normal control |
|  |  | 11.66 | 12.99 | 24.65 | 2 |
|  |  | 11.51 | 12.77 | 24.28 | 3 |
|  |  | 9.74 | 14.34 | 24.08 | 4 |
|  |  | 11.72 | 14.87 | 26.59 | 5 |
| **1.0** | **0** | **9.92** | **13.49** | **23.41** |  | Average |
|  |  | 9.37 | 13.52 | 22.89 | 1 | Diabetic Control |
|  |  | 9.87 | 12.99 | 22.96 | 2 |
|  |  | 9.00 | 13.77 | 22.87 | 3 |
|  |  | 8.11 | 13.36 | 22.85 | 4 |
|  |  | 9.55 | 13.87 | 22.82 | 5 |
| **1.45** | **-0.54** | **9.38** | **13.50** | **22.88** |  | Average |
|  |  | 10.66 | 13.78 | 24.44 | 1 | Diabetic control treated with SM |
|  |  | 10.76 | 13.55 | 24.31 | 2 |
|  |  | 10.97 | 13.45 | 24.42 | 3 |
|  |  | 10.41 | 13.94 | 24.35 | 4 |
|  |  | 11.05 | 13.33 | 24.38 | 5 |
| **0.55** | **0.85** | **10.77** | **13.61** | **24.38** |  | Average |
